# Reemerging Rabies and Lack of Systemic Surveillance in People’s Republic of China

**DOI:** 10.3201/eid1508.081426

**Published:** 2009-08

**Authors:** Xianfu Wu, Rongliang Hu, Yongzhen Zhang, Guanmu Dong, Charles E. Rupprecht

**Affiliations:** Centers for Disease Control and Prevention, Atlanta, GA, USA (X. Wu, C.E. Rupprecht); Academy of Military Medical Science, Changchun, People’s Republic of China (R. Hu); Chinese Centers for Disease Control and Prevention, Beijing, China (Y. Zhang); National Institute for the Control of Pharmaceutical and Biological Products, Beijing (G. Dong)

**Keywords:** Rabies, viruses, carrier-dog phenomenon, China, counterfeit vaccines, seroconversion testing, vaccination coverage, mass vaccination campaigns, postexposure prophylaxis, perspective

## Abstract

Rabies is a reemerging disease in China. The high incidence of rabies leads to numerous concerns: a potential carrier-dog phenomenon, undocumented transmission of rabies virus from wildlife to dogs, counterfeit vaccines, vaccine mismatching, and seroconversion testing in patients after their completion of postexposure prophylaxis (PEP). These concerns are all scientifically arguable given a modern understanding of rabies. Rabies reemerges periodically in China because of high dog population density and low vaccination coverage in dogs. Mass vaccination campaigns rather than depopulation of dogs should be a long-term goal for rabies control. Seroconversion testing after vaccination is not necessary in either humans or animals. Human PEP should be initiated on the basis of diagnosis of biting animals. Reliable national systemic surveillance of rabies-related human deaths and of animal rabies prevalence is urgently needed. A laboratory diagnosis–based epidemiologic surveillance system can provide substantial information about disease transmission and effective prevention strategies.

## Abstract 2

Standardized protocols and diagnostic-based surveillance are imperative for detection and elimination.

The record of rabies in Chinese history dates back to 556 bc in Master Zuo’s tradition of the Spring and Autumn annals. He wrote, “In the eleventh month, people in the capital of Song were chasing a rabid dog. It entered the house of Hua Chen” (*1*). Sporadic descriptions of overt clinical signs of rabies can be found in records of various ancient civilizations (*2*). However, robust scientific investigation of the disease began only after 1885, with Louis Pasteur’s discovery of postexposure vaccination against rabies. In the 1930s, a rabies virus (RABV) 3aG strain was isolated in Beijing and was eventually developed into a vaccine for human immunization. In the 1950s, another RABV strain (CTN) was isolated in Shandong Province and was characterized and attenuated as a vaccine for humans. However, to date, no dog RABV isolates in China have been developed into animal vaccines. Few domestically licensed vaccines for animal rabies exist, according to the Regulations for Veterinary Biologics in China (www.ivdc.gov.cn). The disconnection between human and dog rabies in China reflects a lack of awareness of the concept of one medicine, or health without regard to species, in approaches to rabies control in the public health system.

Although great progress has been made internationally in rabies control and prevention, >55,000 persons still die of rabies annually worldwide. In China, at least 108,412 persons died of rabies from 1950 through 2004 (*3*). A rabies epidemic occurs every 10 years in China (*4*). Despite high human mortality rates, only ≈30 rabies virus isolates have been recorded and partially characterized by sequencing (*3**,**5**,**6*). Therefore, human rabies is mainly reported without confirmatory laboratory diagnosis in most of China. Few statistics are available for dog rabies, indicating that a diagnosis and surveillance system for animal rabies is not fully functional. Obvious inconsistencies exist in published results of human rabies diagnosis (*6*). China is now facing another wave of rabies outbreaks resulting from the combined consequences of rapid economic development, a profitable domestic pet industry, and continuing family planning, resulting in increased numbers of family pets. Reemerging rabies in China has led to a carrier-dog myth, strict pet population control policies, counterfeit vaccines (low antigen, generating <0.5 IU of virus-neutralizing antibodies after administration), vaccine matching, seroconversion testing with an ELISA after completion of postexposure prophylaxis (PEP) in humans, virus–neutralizing antibody titration in vaccinated animals because of inferior vaccines, and other related issues. We discuss these issues and suggest a new approach to prevention and control of rabies when the disease reemerges in an unprepared country like China.

## Carrier or Asymptomatic Rabies

Typically, rabies is fatal once clinical signs develop. Although persistent infections occur regularly for other virus infections, they have not been documented unquestionably in rabies, mainly because of the added complexity of the disease’s relatively long incubation period. The carrier or asymptomatic rabies state was once considered to be important for public health, despite lack of adequate evidence that the phenomenon actually exists. This concern has been raised repeatedly from the early 1930s until recently (*7*). Reported carrier hosts have included vampire bats (*8**,**9*), cats (*10*), dogs (*11**–**15*), and hyenas (*7*). Because rabid dog bites are responsible for ≈99% of all human rabies cases in the world (*16*), the possibility of a carrier state or asymptomatic form of canine rabies deserves serious evaluation. Unfortunately, this possibility remains highly speculative. Although some investigators have questioned reports of a carrier state in dogs (*17*), an author reported RABV isolation from brains of healthy dogs (*6*). Carrier dog RABV isolates were even characterized at the molecular level in 1996 (*15*).

Similarly, reports of healthy-dog carriers were consecutively published in China in 1982 (*18*), 1999 (*19*), 2006 (*20*), and 2007 (*21*). Tang found that 5 (1.76%) of 283 healthy-looking dogs in Guangxi province were positive for RABV by reverse transcription–PCR (RT-PCR) and virus isolation (*5*). Another study showed that 24 of 42 RABV isolates were taken from dogs or cats classified as clinically normal (*3*). All animals from these studies were from areas experiencing rabies epidemics.

All cumulative convincing data for more than half a century from various parts of the world call into question either the concept of a rabies carrier state or the quality of research indicating such a state. Other persistent virus infections routinely occur in lymphocytes, monocytes, macrophages, and dendritic cells through the viruses’ curtailment of the host’s antivirus immune responses. Experimental RABV replication in murine bone marrow macrophages and in human macrophage–like cell lines suggests a mechanism of virus persistence (*22*). However, wild-type RABV is highly neurotropic. If RABV persistent infection occurs in neurons, these findings contradict the current understanding of RABV pathogenesis. Clearly, RABV infection causes minor morphologic changes in neurons but may result in neurophysiologic dysfunction. Also, virus persistence generally is life-long in infected hosts. The longest surviving presumed carrier dog lived for only 16 months; in this dog, the tonsil was found to be the only organ from which the virus was isolated (*14*). No data are available concerning how long such carrier dogs survive. In healthy vampire bats, the duration of salivary excretion of RABV was reported to be 690 days after infection by an extremely high dose of RABV (*8*). Generally, persistent infections are characterized by an excess of viruses or virus antigens; free antibodies, which circulate without binding to antigens, are difficult to detect. However, experimental carrier dogs and vampire bats presented high rates of virus-neutralizing antibodies in serum in these reports (*8**,**14*). From an evolutionary perspective, a carrier dog with normal behavior does not pose an advantage for virus survival because biting when an animal is controlled by an aggressive brain is the only major route for RABV to spread. Animal behavior change is fundamental for RABV survival/transmission. Therefore, if carrier dogs exist, they are inferior to rabid dogs for disease transmission.

In a report by Zhang (*17*), 15 dogs that were diagnosed as positive by ELISA were confirmed to be negative by the standard direct fluorescent antibody (DFA) method. Explanations for these contradictory findings include misidentification of infected dogs, detection of RABV early in the prodromal course once it has reached the central nervous system after infection and incubation, and inadequate diagnostics. These phenomena, rather than the existence of carrier hosts, may explain historical reports of asymptomatic rabies in enzootic areas.

## Rabies Diagnosis in China

A well-established surveillance system for infectious diseases depends on reliable, laboratory-based diagnostic methods. Human and animal rabies cases in China have been reported mainly on the basis of clinical presentations and retrospective epidemiologic surveys. Animal rabies is rarely diagnosed in China. Human rabies diagnosis based solely on clinical symptoms is unreliable because human rabies can be confused with Guillain-Barré syndrome, poliomyelitis, and other types of encephalitis (*23*). Similarly, rabies in animals is difficult to distinguish from canine distemper and other encephalitic conditions. Postmortem rabies diagnosis should be routinely performed on rabid animals, animals that have bitten victims, and human patients who die after an animal bite. Antemortem diagnosis of rabies in humans is challenging because of the disease’s long and variable incubation period. Also, distribution of virus antigens, virus nucleic acids, and antibodies is unpredictable at this stage (*24*). Consequently, all countries should establish standardized national rabies diagnostic protocols for postmortem examinations.

The DFA method, first introduced in the 1950s, is the global standard procedure for rabies diagnosis. It is simple, economical, and reliable (*25*). This method is approved by the World Health Organization (WHO) and the World Organisation for Animal Health (OIE) and has served as a cornerstone for rabies diagnosis for the past half century (*26*). All rabies diagnostic laboratories should follow a single standard protocol (*27*). Whether such a standard exists in China is unclear, and diagnostic reagents, equipment, and qualified diagnosticians are in short supply. The ELISA for RABV antigen detection is carried out in a few laboratories, but this method requires performance evaluation using the standard DFA method to assess specificity, sensitivity, and reproducibility. An ELISA-based rapid rabies enzyme immunodiagnosis (RREID) method that uses monoclonal antibodies against nucleocapsid and glycoprotein for rabies diagnosis has been developed at the Wuhan Institute of Biological Products (*3*). One analysis used the RREID method to detect potential virus antigens in dog saliva; when verified by the DFA method, all 15 samples showed false-negative results (*17*).

Much inconsistency exists when different methods are used for rabies diagnosis (*6*). Overall, of 76 positive samples examined by the DFA method (*6*, cited as an indirect immunofluorescent antibody method), only 36 were confirmed by RT-PCR. In a certified rabies reference laboratory, DFA-positive samples should be positive when confirmed by a sensitive RT-PCR method. Therefore, in China, either the DFA or the RT-PCR, or perhaps both protocols, have questionable validity. The high prevalence of rabies in China necessitates establishment of a standardized national DFA protocol in provincial Centers for Disease Controls and veterinary stations. Other methods should be compared against the established DFA standard. A direct rapid immunohistochemical test (dRIT) using low-cost light microscopy has been extensively investigated and shown to have excellent agreement with the DFA (*28*). The dRIT can be completed within 1 hour, and this method is a feasible alternative at the county level for confirmatory rabies diagnosis or enhanced field surveillance. The urgent need to establish an improved national standard DFA protocol in China should take precedence over current efforts to develop ELISA and RT-PCR methods.

## Rabies Vaccines and Seroconversion Testing in China

From 1885, when the first human rabies vaccination occurred, to 1994, when the RV Street–Alabama-Dufferin (SAD) B19 strain was engineered with reverse genetics (*29*), methods for RABV manipulation have changed fundamentally from random attenuation to defined modifications. However, the basic concept for rabies vaccine development has not changed for more than a century. Several major modern human rabies vaccines include duck embryo vaccine, commercialized in 1957; human diploid cell vaccine, introduced in 1978; purified chicken embryo cell vaccine, developed in 1984; and a purified Vero cell rabies vaccine (PVRV), developed in the late 1980s.

### Human Rabies Vaccines in China

Before the 1980s, nerve tissue-derived Semple vaccine was manufactured using the fixed RABV Beijing strain 3aG, which was isolated in 1931. After the 1980s, primary hamster kidney cells (PHKC) rabies vaccine using the same 3aG strain was investigated as a substitute for nerve tissue vaccines (NTVs) (*30*). In recent years, purified and concentrated Vero cell rabies vaccines using the 3aG and CTN-1 strains have been developed. The PVRV, using a RABV purified Vero (PV) strain imported from the US Centers for Disease Control and Prevention, is also being developed to meet the increasing demand for human rabies vaccine in China. In 2001, WHO issued a resolution for the complete replacement of NTVs by 2006 with cell-culture rabies vaccines. NTVs were gradually replaced by the PHKC vaccine during the 1980s in China.

### Animal Rabies Vaccines in China

In contrast to human rabies vaccine development, animal rabies vaccine development in China has not progressed. In the United States alone, 11 different rabies vaccines are licensed for dogs, 12 for cats, 1 for ferrets, 3 for horses, 4 for cattle, and 5 for sheep (*31*). However, in China, only 1 pentavalent vaccine is licensed, and 1 Flury-low egg passage (LEP) vaccine for dogs has been tentatively approved. No regional RABV isolates were characterized for animal vaccine development. The LEP, Evelyn–Rokitnicki-Abelseth (ERA), PV, and challenge virus standard (CVS) strains being developed as vaccine candidates originate from other countries and have an unclear biological background. The inferior quality of the domestically manufactured dog vaccine in China has been documented (*32*). Consequently, development of animal rabies vaccines using carefully characterized RABV strains should be prioritized as a fundamental task.

Some believe that vaccine production should reflect the design of matched field isolates for regional control. However, vaccine-matching investigations to address concerns about mismatch between vaccine strains and epidemic RABV isolates are redundant (*21*). All fixed RABV strains recommended by WHO, such as PV, CVS, LEP, high egg passage, ERA, and SAD variants, have been successfully used in industrialized countries, where rabies is well controlled. Vaccine quality control and mass production, rather than matching, are urgently needed and most important for addressing the current rabies problem in China. Any potent rabies vaccine will protect against rabies.

### Seroconversion Testing After Vaccination or PEP in China

Because human rabies vaccines in China are produced in cell culture using modern technology, the vaccine quality should follow the standard recommended by WHO. The minimum potency for all cell-culture and purified embryonated egg rabies vaccines is 2.5 IU per intramuscular dose using the National Institutes of Health test (*16*). After rabies exposure in humans, PEP is initiated or withheld based on the postmortem diagnosis of animals that were the source of exposure. Modern PEP is a combination of active and passive immunization, with 100% efficacy in rabies prevention if the process strictly adheres to WHO recommended guidelines (*33**,**34*). Seroconversion testing is not needed for patients completing preexposure or PEP unless the person is immunosuppressed or protocol is not followed (*33*). However, the seroconversion test is routinely performed at the provincial and central levels in China, mainly because of fear of rabies. In animal studies, virus-neutralizing antibody titer has been shown to be an imperfect marker for protection because it varies with time and the expertise of technicians performing the assay. For titration of rabies antibodies, the fluorescent antibody virus neutralization test and the rapid fluorescent focus inhibition test are the only methods approved by WHO and OIE (*35*). However, indirect ELISA or ELISA-based methods are routinely used and are still being developed in China for seroconversion testing after vaccination. Without a standard method, efforts to develop novel assays are a poor use of limited resources for rabies control in China. Potent rabies vaccines do not require routine serologic analysis.

Any failure of vaccination and PEP should be investigated thoroughly and independently to trace potential errors in the protocol. A national vaccine adverse-event reporting system should be established to track suspected problems for safety and efficacy.

## Hosts and Virus Phylogeny

In theory, all mammals are susceptible to RABV infection. In China, dogs play the dominant role in rabies transmission (*4**,**5*). Statistically, >95% of human rabies cases in China are due to rabid dog bites (*16*). In isolated serologic studies of rabies in wildlife such as badgers, raccoon dogs, rodents, and bats, no RABV was successfully isolated from these animals. Rodents do not serve as reservoirs for rabies. In China, all RABV isolates characterized by phylogeny using the N and G gene sequences are categorized into classic RABV genotype 1 (*3**,**6*). Homology among isolates is >90% at the amino acide level. Although some subgroups are suggested, the differences would be minor. In addition, RABV isolates from dogs on all continents are grouped into genotype 1. Phylogeny analyses reinforce the perspective that vaccine matching in China is redundant. In a rabies-epidemic region such as China, rabies in wildlife may result from spillover from dogs. Without proper investigation of animal population density and characterization of the RABV isolates, wildlife rabies in China can be elucidated only after dog rabies is well controlled.

## Interpretation of Rabies in China

The recent reemergence and severe incidence of rabies have attracted the attention of scientists and administrative authorities in China. However, efforts are distracted by and concerns misleadingly focused on healthy dog carriers, possible rabies in wildlife, vaccine matching, inferior or counterfeit vaccines, and seroconversion testing after vaccination. In the 1920s, long before the recognition of bat and other wildlife rabies and the availability of modern vaccines, rabies in Japan was successfully controlled through mass vaccination of dogs. At present, in large cities such as Beijing and Shanghai, although animal bites are fairly common, rabies cases are rare. In 2006, ≈140,000 animal bites were recorded in Beijing, but few human rabies cases were reported (*16*), mainly because of adequate dog vaccination. However, in rural areas of China, dog rabies vaccination coverage is <3% (*4**,**32*). WHO has determined that vaccination coverage >70% is needed to sufficiently control canine rabies, but the exact level of sufficient coverage varies according to demographic, behavioral, and spatial characteristics of dog populations. Historically, the 4 rabies epidemic waves in China reflect discontinuous efforts of dog control and vaccination. The periodic recess of rabies at 10-year intervals resulted from strict depopulation of dogs rather than mass vaccination. The density of 4.5 dogs/km^2^ can lead to endemic rabies in vulnerable populations (*36*). The current estimated number of dogs in China is 80–200 million (*5*). If dogs are distributed evenly, the average density of dogs in China is 8–20/km^2^. The estimated population density would be much higher in rabies-endemic provinces. With very low vaccination coverage in dog populations, rabies outbreaks are not surprising. The phenomenon of 4 rabies epidemic waves during the last half century in China corroborates our interpretation and shows that depopulation of dogs alone cannot efficiently control rabies. Mass vaccination of dogs has been demonstrated to be the most efficient way to control the disease (*37**,**38*).

Decisions to initiate or withhold PEP are based on postmortem diagnosis of the biting animal and use of a standardized method (*27*). Patients exposed to rabies in China were estimated to be between 1% and 10% of the population, according to disease prevalence in different areas. However, because of China’s weak national animal rabies diagnosis network, the number of PEP patients in China is probably arbitrary or only roughly estimated. To maximize efficiency of the limited resource of human vaccines and rabies immunoglobulin for patients at real risk, establishing a systemic rabies diagnosis network is imperative. At a minimum, several million persons are expected to require PEP each year.

RABV is highly neurotropic and functionally conservative. It is transmitted mainly through animal bites, although rare nonbite exposure routes have been reported. Because of this characteristic, the dissemination of rabies is relatively slow. The spread of the disease needs a minimum threshold support of host population density (*36*). Therefore, the frontline of waves of rabies infections garner little public attention. Once the density of dogs reaches a critical threshold, rabies spreads rapidly, and the spread can be accelerated through animal translocation. However, the animal population itself does not pose a rabies threat. An immunized dog population can be a solid barrier to prevent rabies from spreading to humans (*37**,**38*). The immediate challenge for rabies control in China is to stockpile enough vaccines for mass dog vaccination campaigns. A reliable systemic diagnostic network can support effective epidemiologic investigation, vaccine campaigns, and initiation of PEP by providing multiple opportunities for collaborations that work toward practical, humane, and economical rabies elimination in China.
